# Superior Cluneal Nerve Entrapment Syndrome in Adolescents

**DOI:** 10.7759/cureus.80166

**Published:** 2025-03-06

**Authors:** Alvin Jones, Tyler Schimmoeller, Daniele Modderman, Madelyn Hill, Shobhan Vachhrajani

**Affiliations:** 1 Orthopedic Surgery, Cincinnati Children's Hospital Medical Center, Cincinnati, USA; 2 Orthopedic Surgery, Boonshoft School of Medicine, Wright State University, Dayton, USA; 3 Orthopedic Surgery, Dayton Children's Hospital, Dayton, USA

**Keywords:** adolescents, back pain, cluneal nerve, decompression surgery, entrapment syndrome

## Abstract

Objective: Although low back pain is a common symptom in adolescents, the etiology of the pain is frequently unknown. One cause of low back pain that is not widely considered in adolescents and young adults is superior cluneal nerve entrapment syndrome (SCNES). This study describes this disorder in adolescents and young adults and their outcomes after surgical decompression.

Methodology: This is a retrospective case series of 10 patients who underwent surgical decompression of the nerve at a single institution from 2018 to 2020.

Results: All patients with both preoperative and postoperative pain scores reported lower pain scores at their first surgical follow-up. This improvement in postoperative pain scores was statistically significant (P < 0.001). Also, at the latest postoperative follow-up, all patients reported symptom were better than prior to the surgical decompression. One patient required a revision decompression after symptoms recurred. The only complication, postoperative numbness, was reported in two patients. It was unclear whether the numbness was at the incision site or in the distribution of the superior cluneal nerve.

Conclusions: Surgical decompression demonstrated improvement in preoperative pain symptoms in all adolescents in this series with low back pain from SCNES.

## Introduction

Low back pain in adolescents has been reported to be as high as 33% in some studies [1,2]. Determining the etiology of low back pain focuses on structural abnormalities that can be viewed radiographically or with higher-level imaging studies such as computed tomography (CT) scans or magnetic resonance imaging (MRI). Nevertheless, the cause is frequently unidentified [3,4]. Superior cluneal nerve entrapment syndrome (SCNES) is a source of low back pain in adult studies [5,6,7,8]. Specifically, SCNES has been implicated in patients labeled a nonspecific low back pain with an incidence as high as 14% [6,8,9]. It presents with low back pain that has a discrete area of tenderness over the posterior iliac crest 7 centimeters from the midline and radiates from this trigger point area into the buttock and posterolateral thigh region, of which the nerve innervates [7]. Commonly, the pain is aggravated by lumbar hyperextension or crossbody flexion [6]. Pain relief from a trigger point nerve block or corticosteroid injection at the site, 7 cm from the midline, is key to making the correct diagnosis. Nevertheless, if the symptoms recur after the nerve block or corticosteroid injection, then surgical decompression may be considered.

The etiology of SCNES is unknown, and some theorize that it is predisposed by rapid increases in paravertebral muscle tone such as occurs in soldiers in boot camp or young athletes undergoing the pubertal growth spurt [5,10]. It is known to cause associated pain in the buttocks, thighs, and legs in adult patients, mimicking radiculopathy seen in lumbar disk herniations [6,9]. Studies also reveal that the pain is exacerbated by lumbar movements, such as hyperextension, and can mimic clinical signs of spondylolysis [6,7]. Surgical decompression of the entrapped nerve has been reported to be effective in 75%-100% of adults treated for SCNES [5,11].

While SCNES has been highlighted in recent adult studies, there exists a profound lack of awareness of this disorder in the adolescent population. Therefore, clinicians are unlikely to include SCNES in their differential diagnosis of adolescent low back pain. This study presents the largest series of adolescent patients treated for SCNES. This study aims to increase awareness of this disorder by describing the clinical characteristics of affected patients and reporting their surgical outcomes.

## Materials and methods

This is a retrospective case series describing subjects who underwent superior cluneal nerve decompression by a fellowship-trained pediatric orthopedic surgeon at a single institution from January 2018 to December 2021. The institution is a pediatric hospital with age restrictions that limit surgical procedures to patients under 23 years, with an appeals process available for older patients. The appeal process includes a review of the case by pediatric anesthesiologists at the institution who will decide if they are comfortable performing anesthesia for the adult patient. This study population consists primarily of adolescents but also includes some young adults.

Patients included in this study were diagnosed with SCNES using methods previously described in the adult literature. Specifically, the clinical criteria described include low back pain involving the iliac crest and buttock, symptom aggravation with lumbar movement, trigger point tenderness to palpation approximately 7 cm from the midline over the posterior iliac crest, a positive Tinel’s sign, and symptom relief following a nerve block at the trigger point site. The one criterion not verified was a positive Tinel’s sign because this test was not routinely assessed during the clinical exam. All patients included in this analysis had four out of the five criteria present. Patients were excluded from this analysis if they lacked preoperative and/or postoperative pain scores, had less than six months of follow-up data, or underwent concurrent surgery for intervertebral disk herniation. This study received Institutional Review Board approval and an exemption to report these data without consent.

Data collection was conducted retrospectively from the electronic medical records. The data collection tool was created in the Research Electronic Data Capture (REDCap) database. Variables included demographic data, such as age, sex, body mass index (BMI), athletic involvement, previous back trauma, and prior back diagnosis (kyphosis, scoliosis, disc herniation, spondylolysis, spondylolisthesis, compression fracture, etc.). Preoperative and postoperative variables included patient-reported pain scores (pre-and post-operation on a scale of 0-10), characteristics of pain (dull, ache, sharp/stabbing, radiating, localized, burning, crushing), location of pain (from foot to low back), chronicity of pain (<1 year, >1 year) resolution of pain post-op (pain was the same, better, or worse), postoperative complications (numbness, infection, bleeding, etc.), and need for revision procedure (yes, no). The initial postoperative follow-up occurred at approximately four weeks. Subsequently, patients were instructed to follow up at three months, six months, 12 months, and 24 months after surgery.

Frequencies and percentages were used to describe the patient background data and categorical data. For the preoperative and postoperative pain scores, a one-sided paired t-test was performed.

## Results

**Figure 1:**
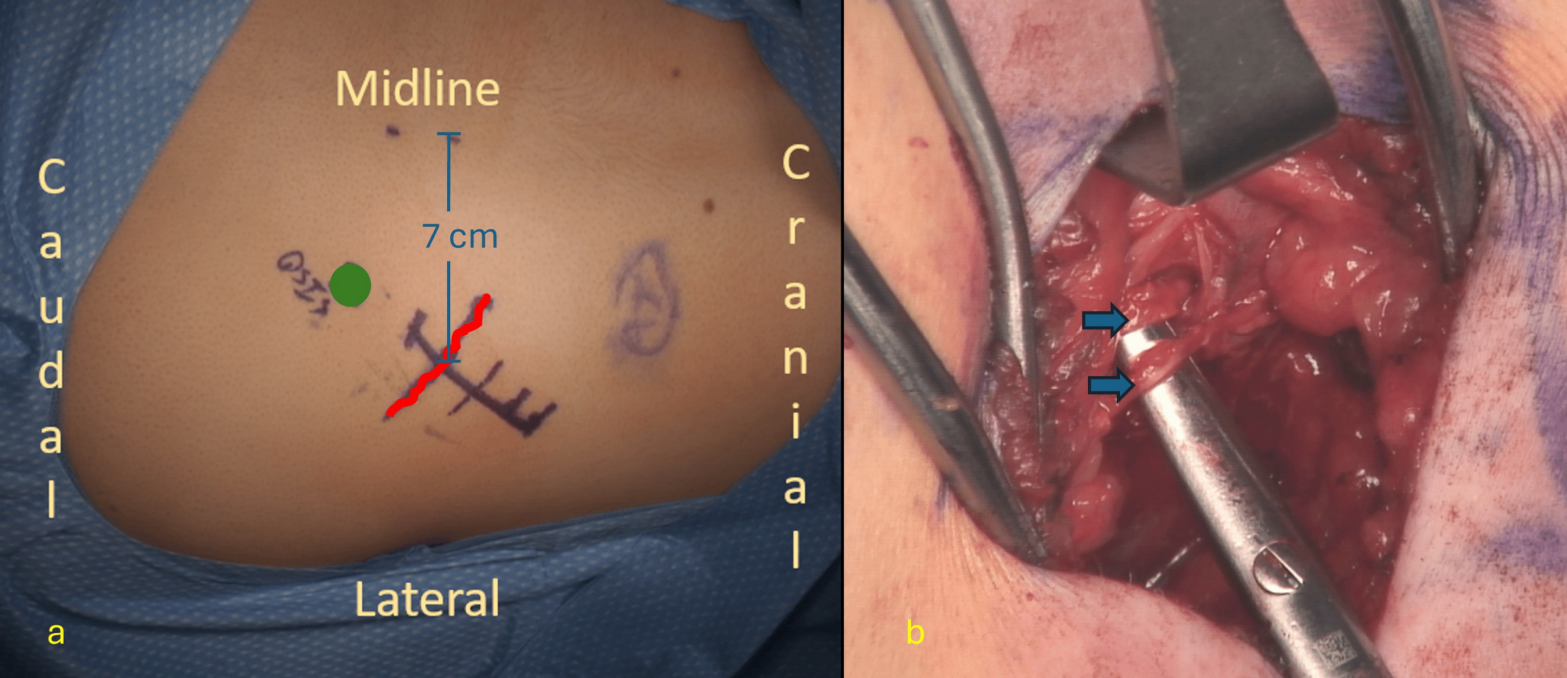
Intra-operative images during a surgical decompression. (a) Anatomic landmarks on the skin before incision. The green circle represents the location of the posterior superior iliac spine. The red squiggly line indicates the expected location of entrapped branches of the superior cluneal nerve where they cross the iliac crest after exiting the thoracolumbar fascia 7 cm from the midline. b: Branches of the superior cluneal nerve after surgical decompression indicated by the blue arrows.

Twenty-four patients met the inclusion criteria. One patient (4%) was excluded due to concurrent surgery for intervertebral disk herniation, seven (29%) were excluded due to a lack of preoperative and/or postoperative pain scores, and another seven (29%) were excluded for having less than six months of follow-up data. Therefore, 10 patients (42%) were included in this analysis. Ages ranged from 13 to 19 years at the time of surgical decompression (Table 1; Figure 1). Most patients were females. BMI varied widely with only three patients in the obese category. No patients indicated a traumatic etiology. Seven had an athletic history, and half presented with a prior diagnosis, all of which were different.

**Table 1:** Demographic and background data. N/A, not applicable; BMI, body mass index

Patient #		Age (years)	Sex	BMI	Athletics	Traumatic etiology	Prior back diagnosis
1	14		F	41	No	N/A	Right snapping hip syndrome
2	19		F	23	Yes	N/A	Lumbar strain
3	16		F	37	No	N/A	N/A
4	17		M	25	Yes	N/A	Sciatica
5	16		F	18	Yes	N/A	N/A
6	17		M	23	Yes	N/A	N/A
7	14		F	30	Yes	N/A	Meralgia paresthetica
8	16		F	19	Yes	N/A	Iliac crest apophysitis
9	13		F	29	Yes	N/A	N/A
10	15		F	21	No	N/A	N/A

Eighty percent reported pain that included the hip and/or thigh (Table 2), and all but one described radiating pain. Six of the patient pain scores were in the moderate range (4-6 out of 10) and the other four were in the severe range (greater than 7). Descriptive pain details were documented for only two patients, who both described a sharp/stabbing sensation. Lastly, six patients described the duration of their pain as greater than one year.

**Table 2:** Preoperative pain data. - indicates no data or missing data.

Patient #	Additional pain locations	Radiating pain	Preoperative pain score	Pain descriptors	Pain duration
1	-	Yes	4	-	<1 year
2	Hip	Yes	5	Sharp, Stabbing	>1 year
3	Thigh	Yes	5	-	<1 year
4	Thigh	Yes	9	-	<1 year
5	-	Yes	7	Sharp, Stabbing	>1 year
6	Hip	Yes	8	-	>1 year
7	Hip	No	7	-	>1 year
8	Hip	Yes	4	-	>1 year
9	Thigh	Yes	4	-	<1 year
10	Hip, Thigh	Yes	5	-	>1 year

Postoperative follow-up ranged from 7 to 18 months, with half of the patients having greater than 12 months of follow-up. Numbness was the only reported postoperative complication noted in two patients (Table 3), and one underwent a revision procedure due to symptom recurrence. At their latest follow-up, all 10 patients reported feeling their pain was better than before surgical decompression. All of these patients showed improvement in their pain scores, which demonstrated statistical significance (Table 4).

**Table 3:** Postoperative results. N/A, not applicable

Patient #	Initial postoperative pain score	Length of follow-up (months)	Complications	Revision needed	Post-revision follow-up time (months)	Reported pain at the latest follow-up
1	1	12	N/A	No	N/A	Better
2	1	8	N/A	No	N/A	Better
3	0	7	N/A	No	N/A	Better
4	3	12	N/A	No	N/A	Better
5	0	18	N/A	No	N/A	Better
6	0	11	N/A	No	N/A	Better
7	0	8	N/A	No	N/A	Better
8	2	17	N/A	Yes	5	Better
9	1	7	Numbness	No	N/A	Better
10	3	15	Numbness	No	N/A	Better

**Table 4:** Preoperative versus postoperative pain scores. T-score = 6.8395. Effect size = 3.06. One-sided paired t-test *P*-value < 0.001.

Patient #	Preoperative pain score	Initial postoperative pain score
1	4	1
2	5	1
3	5	0
4	9	3
5	7	0
6	8	0
7	7	0
8	4	2
9	4	1
10	5	3

## Discussion

This is the largest case series to describe SCNES in this age group. To our knowledge, there have been two case reports describing the existence of SCNES in adolescents [12]. In adult literature, improvement following decompression of the superior cluneal nerve has been reported in 75%-100% of patients [5,11,13]. Our study found similar improvement in this adolescent population. 

All the patients in this study presented after their pubertal growth spurt. To our knowledge, this syndrome has not been described in any prepubescent children. This raises the question of whether there is an association between the adolescent growth spurt and SCNES. Prior studies have suggested an association between SCNES and rapid increases in paravertebral muscle tone, which may also explain the high rate of athletic activity seen in this study [10,5].

Historically, dense fatty nodules at the site of tenderness were thought to cause back pain. These nodules were the focus of earlier treatments and were thought to be fat herniations, *fibrositis*, or lipomas [14,15]. In 1957, Strong reported his results in treating cluneal nerve syndrome and found that only half of his patients had fatty nodules. Therefore, he recommended treating this syndrome with neurectomy and/or neurolysis. Later, in the 1980s and 90s, Maigne and Maigne again published this syndrome as a cause of back pain. They described the anatomy of the superior cluneal nerve and their treatment results [13,16,17,18]. Spine surgeons performing iliac crest bone graft (ICBG) harvesting have been aware of the risk of iatrogenic injury to the superior cluneal nerve [19,20,21]. However, a lack of awareness of non-iatrogenic symptoms arising from this nerve prevents it from being included in the differential diagnosis of patients with chronic lower back pain.

**Figure 2:**
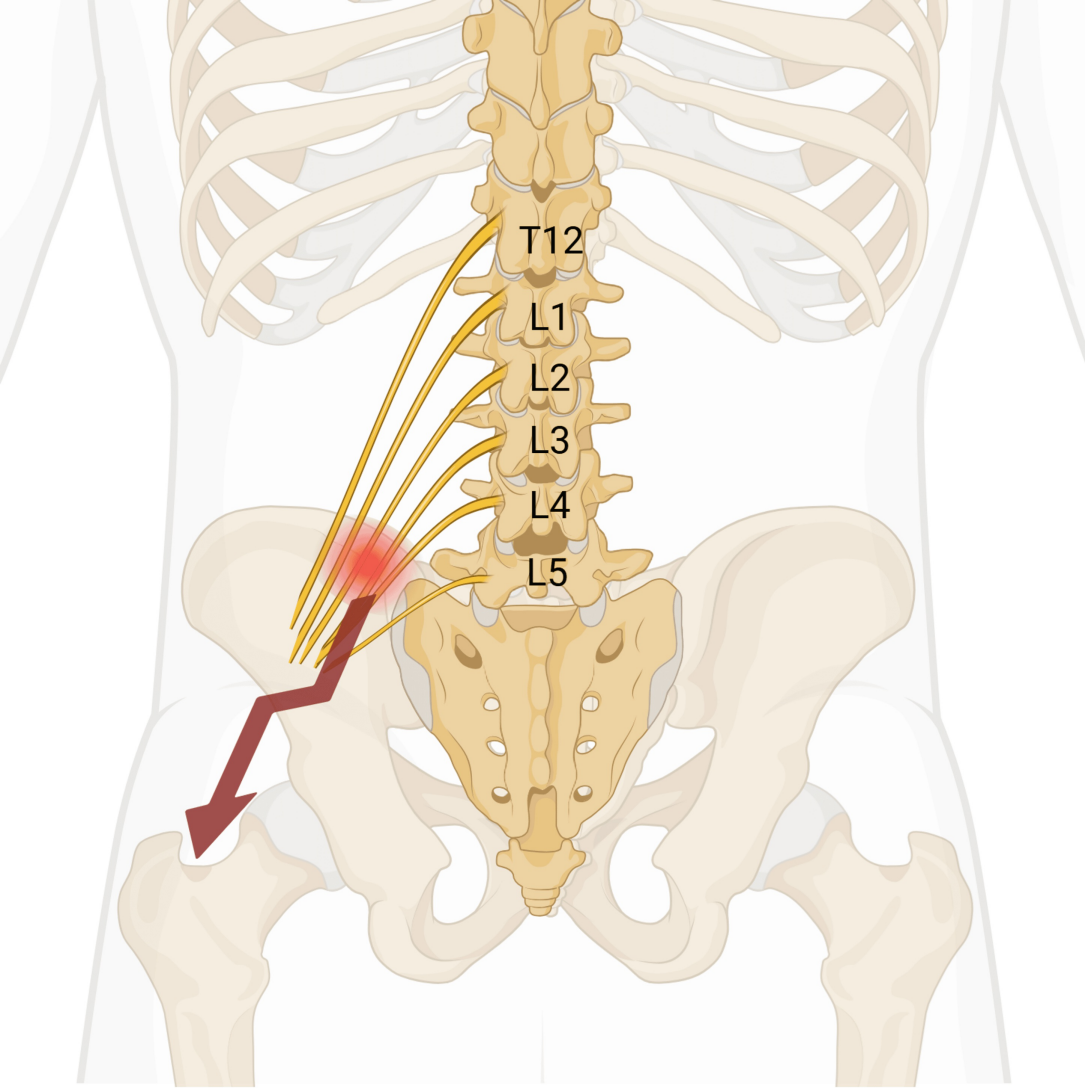
Diagram of the nerve roots (T12-L5) contributing to the branches of the superior cluneal nerve. The red circle indicates the characteristic location where the trigger point pain is located in superior cluneal nerve entrapment syndrome (7 cm from the midline, over the posterior superior iliac crest).  The red arrow indicates the location the pain radiates into the buttocks and posterolateral thigh. Image credit: Created with Biorender.com.

The superior cluneal nerve originates from the T12-L5 nerve roots and can cause radiating pain to the lower extremities as described in this study (Figure 2) [22]. This radiating pain has been termed *pseudo sciatica* and resembles radiculopathy from disk herniation. One study described several adult patients who had previously undergone spinal surgery yet had persistent postoperative pain and were later found to have SCNES [6]. Our study lists the diagnosis patients presented to our clinic with, one of which was sciatica. Also, a more recent report showed an adolescent with chronic low back pain who presented with radiographic evidence of spondylolysis but required only a superior cluneal nerve decompression [23]. There is a concern that in this adolescent population, if SCNES is not considered in the differential diagnosis of chronic low back pain, then patients who fail conservative treatment may end up undergoing more invasive spinal procedures before ruling out this etiology.

We did find one local complication of numbness in two patients, which has not been previously described [24]. One patient underwent a revision around one year out from the initial procedure due to recurrence of symptoms. Dense scar tissue formation at the prior decompression site was seen during the revision procedure.

The retrospective design of this study contributes significantly to its limitations. Due to inconsistent documentation in the electronic medical records, many patients had to be excluded from this analysis, which can inject a form of selection or sampling bias into the study. Also, we found variability documentation at the latest follow-up visits for how patients were describing their current state. For example, some documentation would describe the percentage of pain improvement, others documented less pain, and others would just give a general description of how the patient is doing well or has been able to return to certain activities. Therefore, we coalesced these descriptors into categories of better, worse, or the same as before surgery. This highlights the limitation of our study not having validated low back pain patient-reported outcome measures (PROM) preoperatively and postoperatively to compare results more objectively. 

## Conclusions

In conclusion, this is the largest case series describing the characteristics of adolescents with superior SCNES and their response to surgical decompression. Surgical decompression improved symptoms in all the patients in this series. Numbness was the only postoperative complication described, and one revision was performed due to dense scar tissue. Future prospective studies would benefit from standardized data collection and validated patient-reported outcome measures for low back pain. Also, the consideration of methods to prevent the formation of dense scar formation should be explored as a possible way to prevent the need for revision procedures.

## References

[1] Jeffries LJ, Milanese SF, Grimmer-Somers KA (2007). Epidemiology of adolescent spinal pain: a systematic overview of the research literature. Spine (Phila Pa 1976).

[2] Calvo-Muñoz I, Gómez-Conesa A, Sánchez-Meca J (2013). Prevalence of low back pain in children and adolescents: a meta-analysis. BMC Pediatr.

[3] Deyo RA, Weinstein JN (2001). Low back pain. N Engl J Med.

[4] Miller R, Beck NA, Sampson NR, Zhu X, Flynn JM, Drummond D (2013). Imaging modalities for low back pain in children: a review of spondyloysis and undiagnosed mechanical back pain. J Pediatr Orthop.

[5] Strong EK, Davila JC (1957). The cluneal nerve syndrome; a distinct type of low back pain. Ind Med Surg.

[6] Kuniya H, Aota Y, Kawai T, Kaneko K, Konno T, Saito T (2014). Prospective study of superior cluneal nerve disorder as a potential cause of low back pain and leg symptoms. J Orthop Surg Res.

[7] Iwamoto N, Isu T, Kim K (2016). Low back pain caused by superior cluneal nerve entrapment neuropathy in patients with Parkinson disease. World Neurosurg.

[8] Talu GK, Ozyalçin S, Talu U (2000). Superior cluneal nerve entrapment. Reg Anesth Pain Med.

[9] Isu T, Kim K, Morimoto D, Iwamoto N (2018). Superior and middle cluneal nerve entrapment as a cause of low back pain. Neurospine.

[10] Ermis MN, Yildirim D, Durakbasa MO, Tamam C, Ermis OE (2011). Medial superior cluneal nerve entrapment neuropathy in military personnel; diagnosis and etiologic factors. J Back Musculoskelet Rehabil.

[11] Morimoto D, Isu T, Kim K, Imai T, Yamazaki K, Matsumoto R, Isobe M (2013). Surgical treatment of superior cluneal nerve entrapment neuropathy. J Neurosurg Spine.

[12] Aly TA, Tanaka Y, Aizawa T, Ozawa H, Kokubun S (2002). Medial superior cluneal nerve entrapment neuropathy in teenagers: a report of two cases. Tohoku J Exp Med.

[13] Maigne JY, Doursounian L (1997). Entrapment neuropathy of the medial superior cluneal nerve. Nineteen cases surgically treated, with a minimum of 2 years' follow-up. Spine (Phila Pa 1976).

[14] CO WS, AC WL (1947). Edema or herniations of fat lobules as a cause of lumbar and gluteal fibrositis. Arch Intern Med (Chic).

[15] Reis E (1937). Episacroiliac lipoma. Am J Obstetr Gynecol.

[16] Maigne R (1980). Low back pain of thoracolumbar origin. Arch Phys Med Rehabil.

[17] Maigne JY, Lazareth JP, Guérin Surville H, Maigne R (1989). The lateral cutaneous branches of the dorsal rami of the thoraco-lumbar junction. An anatomical study on 37 dissections. Surg Radiol Anat.

[18] Maigne JY, Maigne R (1991). Trigger point of the posterior iliac crest: painful iliolumbar ligament insertion or cutaneous dorsal ramus pain? An anatomic study. Arch Phys Med Rehabil.

[19] Banwart JC, Asher MA, Hassanein RS (1995). Iliac crest bone graft harvest donor site morbidity. A statistical evaluation. Spine (Phila Pa 1976).

[20] Fernyhough JC, Schimandle JJ, Weigel MC, Edwards CC, Levine AM (1992). Chronic donor site pain complicating bone graft harvesting from the posterior iliac crest for spinal fusion. Spine (Phila Pa 1976).

[21] Kurz LT, Garfin SR, Booth RE Jr (1989). Harvesting autogenous iliac bone grafts. A review of complications and techniques. Spine (Phila Pa 1976).

[22] Konno T, Aota Y, Kuniya H (2017). Anatomical etiology of "pseudo-sciatica" from superior cluneal nerve entrapment: a laboratory investigation. J Pain Res.

[23] Ruan T, Jones AC (2023). Superior cluneal nerve entrapment syndrome: thought to be spondylolysis. J Am Acad Orthop Surg Glob Res Rev.

[24] Najjar E, Karouni F, Komaitis S, Boszczyk B, Quraishi NA (2023). Cluneal nerve release: a systematic review. Eur Spine J.

